# Supplementary material for: Risk Perception, Protective Behaviors, and General Anxiety during the Coronavirus Disease 2019 Pandemic among Affiliated Health Care Professionals in Taiwan: Comparisons with Frontline Health Care Professionals and the General Public
Source: Int J Environ Res Public Health. 2020 Dec 13;17(24):9329. doi: 10.3390/ijerph17249329 (PMC7764763; doi:10.3390/ijerph17249329)
Supplement: Supplementary file 1 [file ijerph-17-09329-s001.pdf]

International Journal of
Environmental Research and Public Health

MDPI

Supplementary Table S1. Measures used in this study.

<table>
<tr><th>Measures</th><th>Items</th><th>Response Scale</th><th colspan="2">Scoring</th></tr>
<tr><td rowspan="5">Risk perception of COVID-19</td><td>Item 1: If you were to develop flu-like symptoms tomorrow, would you worry?</td><td>1 = not at all worried, 2 = worried less than normal, 3 = about the same, 4 = worried more than normal, 5 = extremely worried</td><td>Divided by 5</td><td rowspan="5">Summed to obtain a score</td></tr>
<tr><td>Item 2: In the past one week, have you ever worried about catching COVID-19?</td><td>1 = no, never think about it, 2 = think about it but it didn't worry me, 3 = worried me a bit, 4 = worried me a lot, 5 = worried about it all the time</td><td>Divided by 5</td></tr>
<tr><td>Item 3: Please rate the current level of your worry towards <u>COVID-19</u>:</td><td>Score ranged from 1–10 (1 = very mild, 10 = very severe)</td><td>Divided by 10</td></tr>
<tr><td>Item 4: How likely do you think it is that you will contract COVID-19 over the next 1 month?</td><td>1 = never, 2 = very unlikely, 3 = unlikely, 4 = evens, 5 = likely, 6 = very likely, 7 = certain</td><td>Divided by 7</td></tr>
<tr><td>Item 5: What do you think are your chances of getting COVID-19 over the next 1 month compared to others outside your family?</td><td>1= not at all, 2 = much less, 3 = less, 4 = evens, 5 = more, 6 = much more, 7 = certain</td><td>Divided by 7</td></tr>
<tr><td>Adoption of protective behaviors</td><td>In the past week, did you 1) avoid going to crowded places, 2) wash your hands more often, 3) wear a mask more often, 4) maintain good indoor ventilation, and 5) disinfecting household frequently?</td><td>0 = no, 1 = yes, but not due to COVID-19, 2 = yes, due to COVID-19</td><td>0 = no (score ≤1)
1 = yes (score = 2)</td><td>Summed to obtain a score</td></tr>
<tr><td>General anxiety</td><td>Agreement on 10 statements: You feel rested /content/comfortable/relaxed/pleasant/anxious/nervous /jittery/"high strung"/over-excited and "rattled"</td><td>1 = not at all, 2 = sometimes, 3 = moderately so, 4 = very</td><td colspan="2">Summed to obtain a score</td></tr>
</table>
